# CsWAKL08, a pathogen-induced wall-associated receptor-like kinase in sweet orange, confers resistance to citrus bacterial canker via ROS control and JA signaling

**DOI:** 10.1038/s41438-020-0263-y

**Published:** 2020-04-01

**Authors:** Qiang Li, Anhua Hu, Jingjing Qi, Wanfu Dou, Xiujuan Qin, Xiuping Zou, Lanzhen Xu, Shanchun Chen, Yongrui He

**Affiliations:** Citrus Research Institute, Southwest University/Chinese Academy of Agricultural Sciences, Chongqing, 400712 China

**Keywords:** Plant physiology, Plant stress responses

## Abstract

Citrus bacterial canker (CBC) is a disease resulting from *Xanthomonas citri* subsp. *citri* (Xcc) infection and poses a grave threat to citrus production worldwide. Wall-associated receptor-like kinases (WAKLs) are proteins with a central role in resisting a range of fungal and bacterial diseases. The roles of WAKLs in the context of CBC resistance, however, remain unclear. Here, we explored the role of CsWAKL08, which confers resistance to CBC, and we additionally analyzed the molecular mechanisms of CsWAKL08-mediated CBC resistance. Based on systematic annotation and induced expression analysis of the CsWAKL family in *Citrus sinensis*, CsWAKL08 was identified as a candidate that can be upregulated by Xcc infection in the CBC-resistant variety. CsWAKL08 can also be induced by the phytohormones salicylic acid (SA) and methyl jasmonic acid (MeJA) and spans the plasma membrane. Overexpression of CsWAKL08 resulted in strong CBC resistance in transgenic sweet oranges, whereas silencing of CsWAKL08 resulted in susceptibility to CBC. The peroxidase (POD) and superoxide dismutase (SOD) activities were significantly enhanced in the CsWAKL08-overexpressing plants compared to the control plants, thereby mediating reactive oxygen species (ROS) homeostasis in the transgenic plants. Moreover, the JA levels and the expression of JA biosynthesis and JA responsive genes were substantially elevated in the CsWAKL08 overexpression plants relative to the controls upon Xcc infection. Based on these findings, we conclude that the wall-associated receptor-like kinase CsWAKL08 positively regulates CBC resistance through a mechanism involving ROS control and JA signaling. These results further highlight the importance of this kinase family in plant pathogen resistance.

## Introduction

Plants have evolved a plethora of strategies to defend against microbial pathogens, including bacteria, viruses, and fungi^1^. Some of the most readily engaged defense mechanisms depend on rapid pathogen recognition via directly recognizing either pathogen-associated molecular patterns (PAMPs) or damage-associated molecular patterns (DAMPs) using a series of genetically encoded innate immune receptors expressed on the plasma membrane^2,3^. PAMPs are often highly conserved microbial structures, and thus, these responses in plants result in broad resistance to entire pathogen classes^4–6^. In contrast, certain receptor kinases can provide plants with resistance to just one or a limited subset of pathogenic microbes^7^. Receptor-like kinases (RLKs) are a broad class of receptor kinases, exhibiting a wide range of extracellular motifs well suited for the recognition of PAMPs/DAMPs, including leucine-rich repeats, lectin and lysine motifs, and epidermal growth factor-like extracellular domains^5,6^. RLKs are key mediators of plant innate immunity, recognizing and inducing specific intracellular signals in response to PAMPs as a part of specific plasma membrane protein complexes^8^. RLKs then activate plant immunity through the activation of transcription factors and through the regulation of reactive oxygen species (ROS) homeostasis, pathogenesis-related (PR) proteins, and phytohormones^9,10^. Phytohormones, including jasmonic acid (JA), salicylic acid (SA) and abscisic acid (ABA), are essential mediators of plant immunity and play major roles in the regulation of plant defense responses^11^.

In vascular plants, wall-associated kinases (WAKs) containing a cell wall-associated galacturonan-binding domain^12^ are an RLK subfamily present in association with the plant cell wall^13^. An initial study of *Arabidopsis thaliana* first identified 5 WAK genes (*WAK1*-*WAK5*) and 22 WAK-like genes (*WAKL1*-*WAKL22*)^14,15^. These WAK(L)s are the only proteins that are known to serve as a direct link between the cell wall and the plasma membrane, thus mediating rapid intracellular signal transduction in response to the activation of their extracellular receptor-like domains^13,16,17^. WAK-like kinases (WAKLs) have been recently found to be essential mediators of innate resistance to specific bacterial and fungal pathogens in a wide range of cereal plant species. For example, *qHSR1*, *Htn1*, and *Xa4* are three such genes involved in conferring resistance to maize head smut, maize northern corn leaf blight and rice bacterial blight diseases, respectively^3,7,18^. Resistance mechanisms engaged in response to WAK signaling include increased cellulose and phytoalexin synthesis to bolster cell wall integrity^7^, reconstructed homeostasis of ROS species including hydrogen peroxide (H_2_O_2_) and superoxide (O_2_^•^−)^19^, and upregulation of specific pathogen defense genes^18^. While the WAK and WAKL genes are most typically associated with disease resistance, this is not universally true. For example, in wheat, the *Snn1 WAK* senses and triggers cell death in response to the SnTox1 toxin produced by the *Parastagonospora nodorum* fungus, thereby mediating fungal proliferation and conferring disease susceptibility^20^.

Citrus bacterial canker (CBC) is a quarantine-requiring disease caused by the infection of *Xanthomonas citri* subsp. *citri* (Xcc), leading to substantial losses in citrus yields globally^21–24^. We previously found in long-term analyses of Xcc-induced citrus transcriptomic changes that WAKLs were highly represented in the differentially expressed genes (DEGs) (unpublished data). These results led us to explore the relationship between CBC and WAKLs, which may be potential candidate genes for CBC resistance breeding. As plant genome sequences are increasingly available, the WAKL families of several plants have been annotated and thoroughly researched^25^. However, to date, no systematic study of WAKLs has been performed in *Citrus* species. High-quality sweet orange genome data offer an ideal opportunity to conduct a genome-wide analysis of citrus WAKLs (CsWAKLs)^26^.

Here, we conducted a comprehensive assessment of the identities and functions of CsWAKLs in response to CBC. The roles of CsWAKLs in CBC resistance were investigated via overexpression and RNAi silencing approaches, and the mechanisms of CsWAKL-mediated CBC resistance were next assessed in transgenic plants by several physiological and biochemical analyses. Finally, we found that CsWAKL08 plays an important role in the interference of Xcc pathogenesis in sweet orange by the JA signaling pathway and ROS homeostasis, providing evidence that CsWAKL08 is directly involved in the defense responses to Xcc.

## Results

### Identification and bioinformatic analysis of the CsWAKLs in sweet orange

**Fig. 1:**
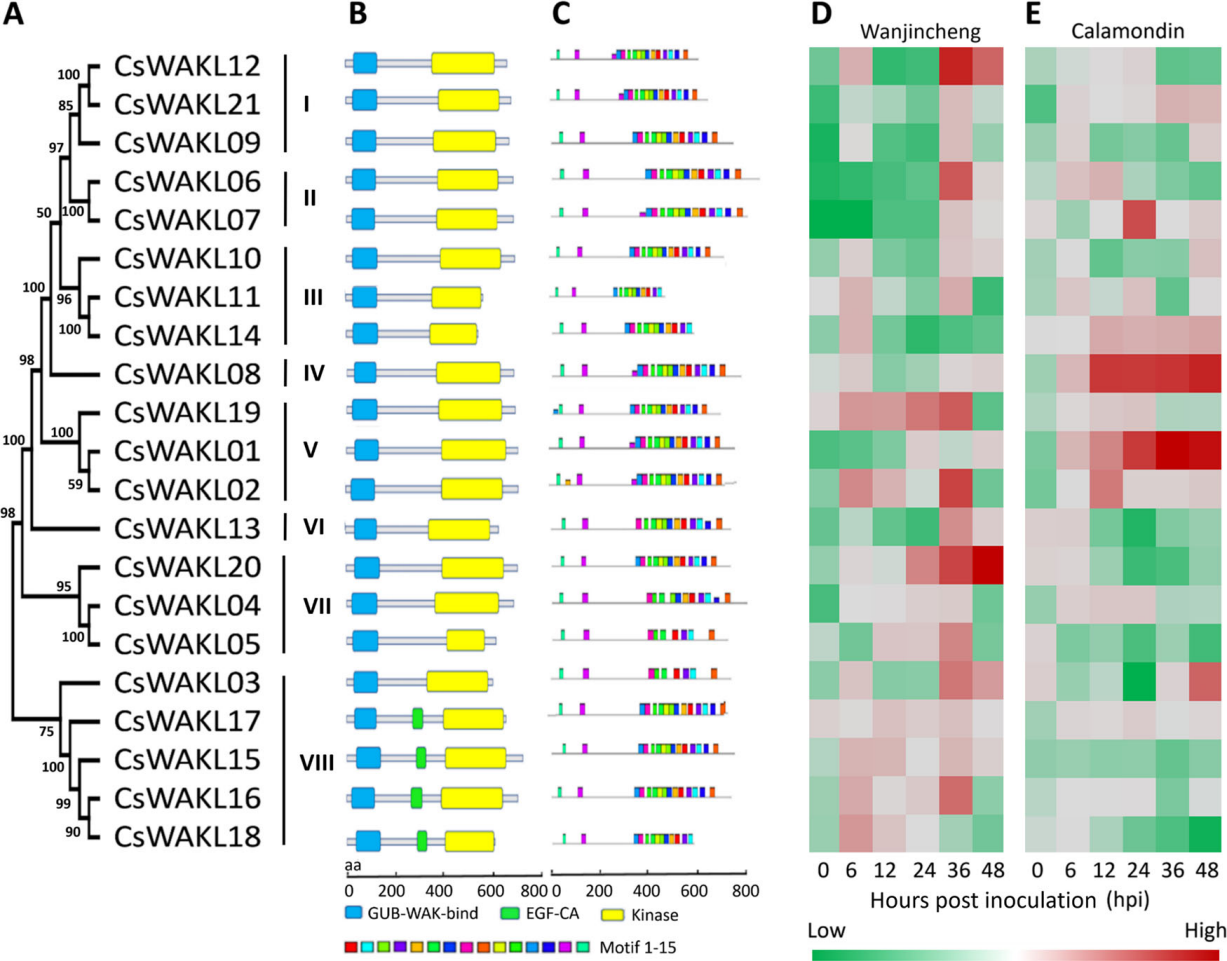
Genome-wide annotation and Xcc-induced expression profiling of the CsWAKL family. **a** Phylogeny of CsWAKLs. The full-length protein sequences were used for the neighbor-joining tree with MEGA V7.2 based on a bootstrap model (bootstrap = 100). **b** The functional domains of 21 CsWAKLs as predicted using Pfam V32. Different domains are represented by differently colored rounded rectangles. **c** The conserved motifs of CsWAKLs as determined using MEME V5.1. Different expression profiles of CsWAKLs induced by Xcc in Wanjincheng (**d**) and Calamondin (**e**) were detected via qRT-PCR with CsActin (GenBank: GU911361.1) as a normalization control. Samples were collected at 0–48 hpi (hours post inoculation). In the heatmap, green and red correspond to low and high expression levels, respectively

Based on exhaustive data mining and a semiautomatic annotation protocol, we identified 21 *WAKLs* from the genome of *C. sinensis*, which were designated *CsWAKL01* to *CsWAKL21* according to their chromosomal locations (Table S1). Compared with that in monocotyledonous plants such as rice and maize, which possess more than 100 *WAKLs*, the WAKL gene family in dicotyledonous plants is relatively small (*C. sinensis*: 21 and *A. thaliana: 27*)^18,27^. To investigate the evolutionary relationships among the WAKL family members in *C. sinensis*, we constructed a phylogenetic tree of all 21 CsWAKLs using the neighbor-joining method with MEGA V7.2^28^. Based on the phylogenetic relationships of the CsWAKLs, these 21 members can be divided into eight groups containing six clusters and two singletons (CsWAKL08 and CsWAKL13) with strong bootstrap values (Fig. 1a).

With respect to functional annotation, each CsWAKL possesses an N-terminal GUB-WAK domain (Pfam: 13947) and a C-terminal kinase domain (Pfam: 00069). Four CsWAKLs (CsWAKL15–18) also have a calcium-binding EGF-CA domain (Pfam: 07645) following the GUB-WAK domain (Fig. 1b). Conserved motif analyses of these CsWAKLs were performed to identify their functional regions. In total, 15 conserved motifs with 6–20 residues in these 21 complete CsWAKLs were detected using MEME V5.1^29^. The motif composition and arrangements are consistent with the phylogenetic tree. The conserved motifs are not evenly distributed along the CsWAKLs. The N-terminal regions of these 21 CsWAKLs are unique, whereas the C-terminus is more conserved (Fig. 1c).

### CsWAKL08 is a putative Xcc-influenced CBC resistance gene

To assess the function of CsWAKL, we examined the expression of these 21 CsWAKLs in the context of biotic stress. To identify the relationships between CsWAKLs and CBC, we detected the expression of the CsWAKLs between 0 and 48 hours post inoculation (hpi) by quantitative real-time polymerase chain reaction (qRT-PCR) in the CBC-sensitive variety Wanjincheng (Fig. 1d) and in the CBC-resistant variety Calamondin (Fig. 1e). These 21 CsWAKLs exhibited various expression profiles in response to Xcc infection (Fig. S1). In Calamondin, 2 CsWAKLs (CsWAKL01 and CsWAKL08) were upregulated continuously, whereas in Wanjincheng, CsWAKL01 and CsWAKL08 expression did not change significantly during Xcc infection. These two CsWAKL genes may thus represent Xcc resistance genes. CsWAKL20, in contrast, exhibited an expression profile opposite that of CsWAKL01 and CsWAKL08, increasing in Wanjincheng while decreasing in Calamondin. This gene may thus be linked to Xcc susceptibility. In summary, our comprehensive annotation efforts and analyses of Xcc-induced expression changes led us to identify CsWAKL08, CsWAKL01, and CsWAKL20 as the best candidates for further CBC research, and we focused on CsWAKL08 hereafter.

### *CsWAKL08* encodes a WAKL

**Fig. 2:**
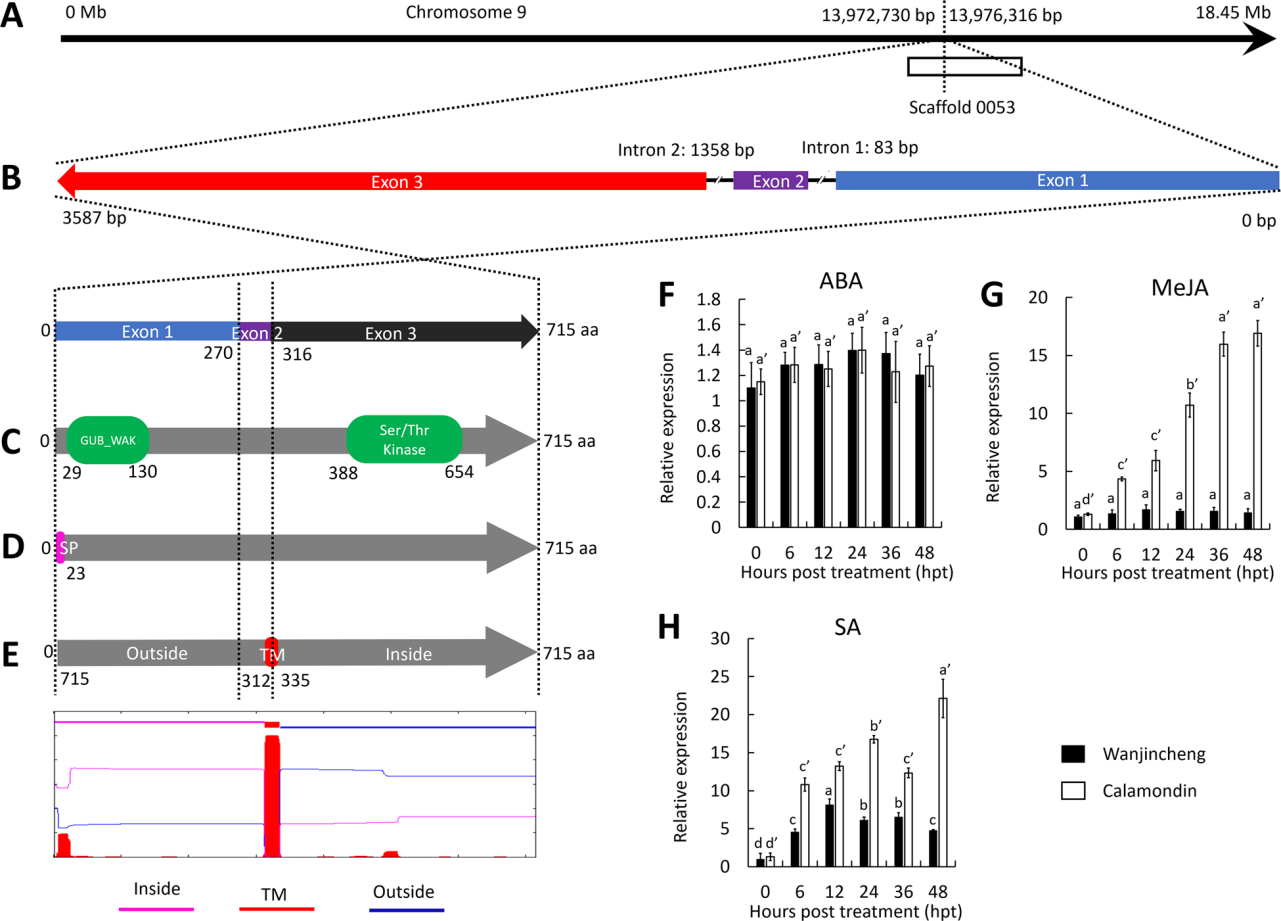
Bioinformatics and expression characteristics of CsWAKL08. **a** The chromosomal locus of CsWAKL08. Data were retrieved from CAP. **b** The exon-intronic structures of *CsWAKL08* determined using GSDS V2.0. **c** The functional domains of CsWAKL08 determined using Pfam V32. **d** The CsWAKL08 signal peptide prediction conducted using SignalP V4.0. **e** Illustration of the putative TM region of CsWAKL08 predicted by TMHMM V2.0. The expression profiles induced by ABA (**f**), MeJA (**g**) and SA (**h**) were quantified via qRT-PCR with CsActin (GenBank: GU911361.1) as a normalization control. Samples were collected at 0–48 hpt (hours post treatment). In (**f**)–(**h**), Tukey’s honestly significant difference (*P* = 0.05) test was used to analyze the data, with three biological replicates per sample; data are the mean ± SD

We obtained the complete transcript sequence of CsWAKL08 using RNA isolated from Wanjincheng leaf samples. The sequence was consistent with the sequence assembled in the Citrus Annotation Project (CAP)^26^. CsWAKL08 is a 715-residue WAKLs encoded by a gene located on chromosome 9 of *C. sinensis*, with ORFs likely to encode functional proteins^26^ (Fig. 2a). It is encoded on the minus strand and contains a short intron (intron 1; 83 bp) and a 1358-bp intron (intron 2) as determined based on an exon-intronic survey (Fig. 2b). The N-terminal domain is a cysteine-rich galacturonan-binding region (GUB-WAK) from aa 29 to aa 130, encoded by exon 1 (Fig. 2c). This cysteine-rich domain is characteristic of WAKs^15^. The C-terminal domain contains a cytoplastic Ser/Thr kinase domain (aa 388 to aa 654, encoded by exon 3) (Fig. 2c), which is central for its kinase activity. At the N-terminus of CsWAKL08, a 23-aa signal peptide (SP) was detected, consistent with trafficking to the extracellular environment (Fig. 2d). Furthermore, the protein contains a predicted transmembrane (TM) structure between aa 312 and aa 335 that is encoded by both exons 2 and 3 (Fig. 2e). These features are all consistent with the WAKL identity of CsWAKL08.

### CsWAKL08 was induced by SA and MeJA

Phytohormones commonly regulate the expression of plant disease-associated proteins^18,21^. To examine the role of CsWAKL08 in disease resistance-related signaling, we performed ABA, MeJA and SA stimulation, and CsWAKL08 expression was analyzed via qRT-PCR. The results indicated that upon ABA treatment, the expression of CsWAKL08 was not significantly altered during the 48-h treatment period in either the Calamondin or Wanjincheng plants (Fig. 2f). However, CsWAKL08 expression in Calamondin upon exogenous MeJA treatment was sharply elevated (14-fold), whereas no significant changes in expression were detected in Wanjincheng (Fig. 2g). With respect to SA induction, the expression of CsWAKL08 was also increased and maintained at high levels in Calamondin within the 48-h treatment period. CsWAKL08 expression significantly increased in Wanjincheng within the first 12-h post SA treatment and then decreased continuously thereafter (Fig. 2h). Based on these results, we concluded that in the CBC-resistant variety Calamondin, CsWAKL08 expression can be induced by both MeJA and SA, whereas in Wangjincheng, CsWAKL08 expression can be induced to a lesser degree. These findings indicate that CsWAKL08 plays a role in some disease resistance-associated signaling pathways.

### CsWAKL08 localizes to the plasma membrane

**Fig. 3:**
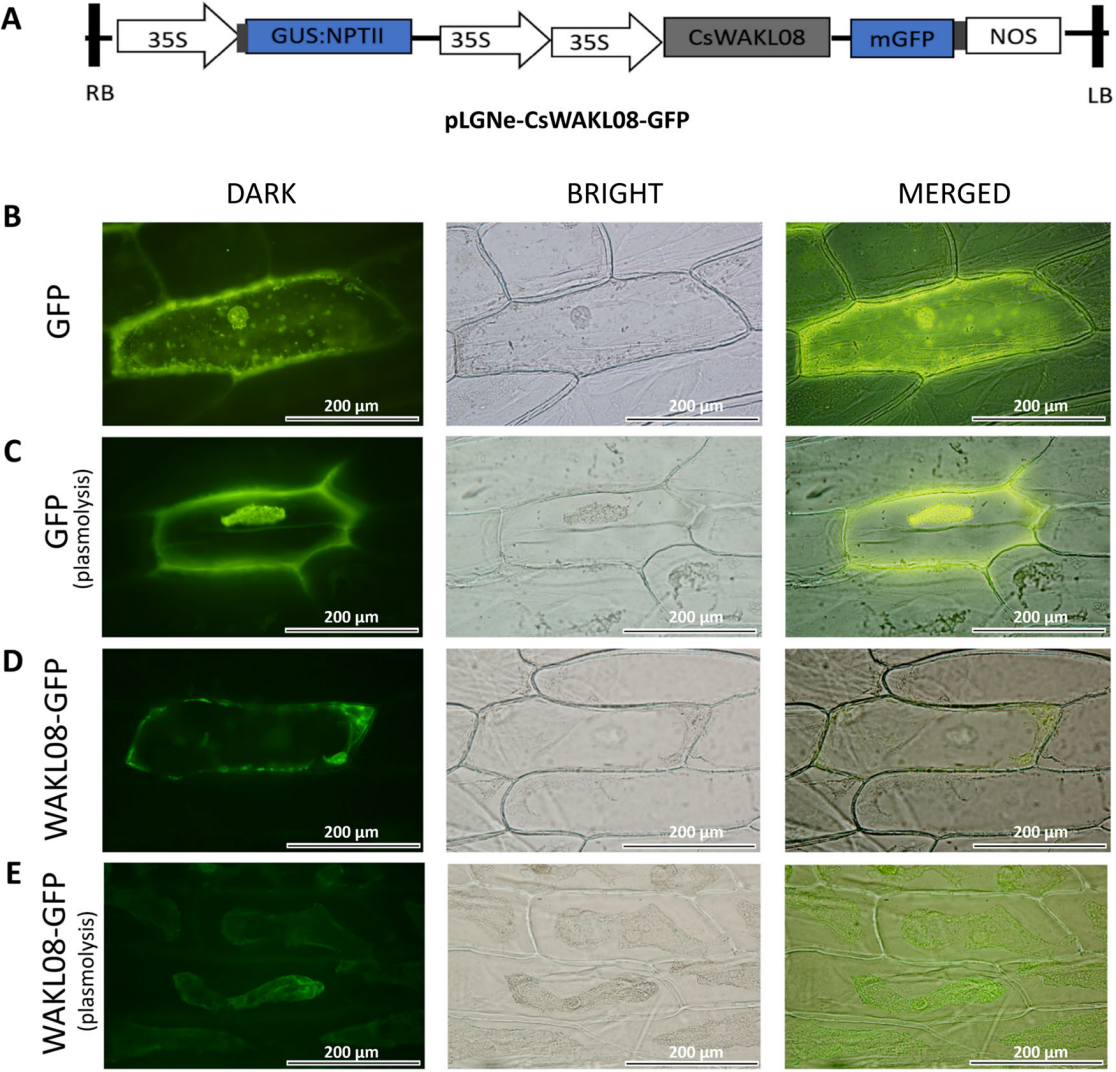
CsWAKL08 localizes to the plasma membrane. **a** The plasmid used for transient expression; 35S, cauliflower mosaic virus 35S promoter; NOS, NOS terminator; NPTII, NptII gene; GFP, GFP gene; LB: left border; RB: right border. GFP fluorescence of baseline onion epidermal cells (**b**) and after plasmolysis (**c**) were taken as the control. Transient expression was measured based on the fluorescence of CsWAKL08-GFP at baseline (**d**) and after plasmolysis (**e**). In (**b**)–(**e**), scale bar = 200 μm; fields of view are shown as dark field, bright field, and merged images

To establish the subcellular localization of CsWAKL08, we employed both predictive analyses and a transient expression system. CELLO showed that the extracellular locus value for this protein of 2.98, which was larger than other locus values, suggesting that CsWAKL08 was an extracellular protein (Table S2). To validate this prediction, we assessed CsWAKL08 localization within cells after transiently expressing the recombinant pLGNe-CsWAKL08-GFP plasmid (Fig. 3a). In the control, both the nucleus and cytoplasm exhibited green fluorescence, as indicated via microscopic examinations before and after plasmolysis (Fig. 3b, c). In contrast, CsWAKL08-GFP was strongly evident in the plasma membrane of epidermal onion cells before and after plasmolysis (Fig. 3d, e), similar to the ZmWAK-GFP signal reported previously^18^. Both predictive analyses and transient expression thus clearly indicated that CsWAKL08 is a cell membrane-localized protein that can play a role in extracellular signal reception.

### Overexpression of CsWAKL08 confers CBC resistance

**Fig. 4:**
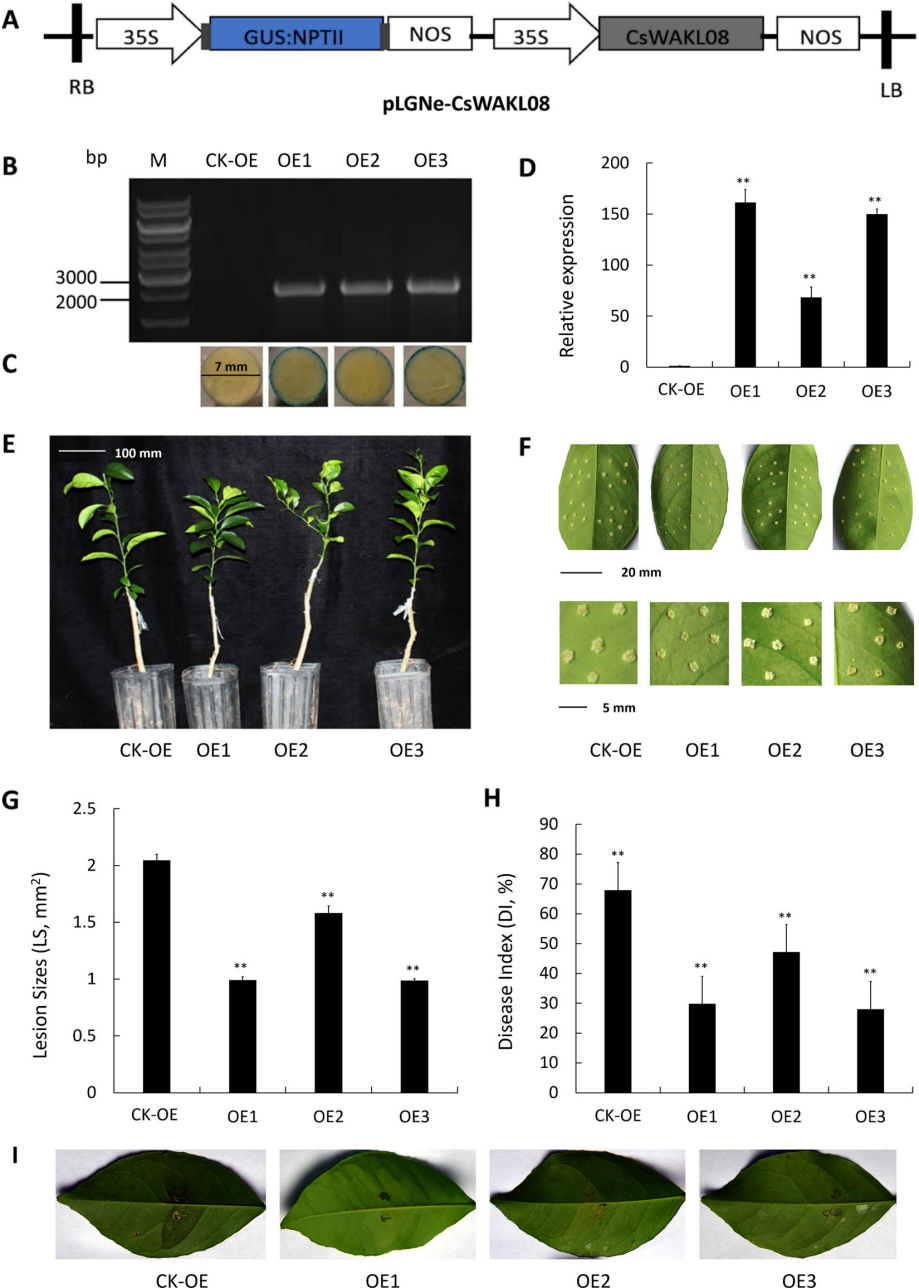
Assessment of Xcc responses in the CsWAKL08-overexpressing plants. **a** Recombinant plasmid used for overexpression assays; 35S, cauliflower mosaic virus 35S promoter; NOS, NOS terminator; NPTII, NptII gene; LB: left border; RB: right border. Validation of the transgenic plants by PCR (**b**) and by GUS staining for 24 h (**c**). **d** CsWAKL08 expression in the overexpression plants as assessed via qRT-PCR with CsActin (GenBank: GU911361.1) as a normalization control. **e** Phenotypes of the transgenic plants. Scale bar = 100 mm. **f** Xcc-induced disease symptoms on the control and transgenic plants inoculated with Xcc at 10 dpi. The scale bar for the leaves was 20 mm, while for the lesions, it was 5 mm. For analysis of disease resistance, lesion sizes (LS) (**g**) and disease index (DI) (**h**) were analyzed. In (**d**), (**g**) and (**h**), **P < 0.01, extremely significant difference; data are the mean ± SD. **i** CBC resistance assay of overexpression plants using the infiltration method. Xcc-induced disease symptoms were observed at 10 dpi

Transgenic citrus constructs that overexpress CsWAKL08 were next used to further elucidate the role of CsWAKL08 and to further examine its role in Xcc resistance. A CsWAKL08 overexpression vector with a GUS coding sequence driven by a CaMV 35S promoter was constructed for this study (Fig. 4a). A fragment (2688 bp) was detected upon PCR in three transgenic plants (OE1–OE3) that was absent in the control plants (CK) transformed with the empty vector (Fig. 4b), and blue color was evident on the leaf disc edge by GUS staining (Fig. 4c). qRT-PCR further confirmed that these three overexpression plants expressed high levels of CsWAKL08 (161-fold, 68-fold, and 149-fold over CK, respectively) (Fig. 4d). With respect to their phenotypes, the three transgenic plants exhibited a normal growth rate compared to the CK plants (Fig. 4e). To study the CBC resistance of these OE1, OE2, and OE3 transgenic plants, we performed an in vitro assay via pinprick inoculation. Overexpression transgenic leaves exhibited significantly smaller lesions and exhibited less serious symptoms than those of the CK plants (Fig. 4f). The Xcc pustules were alleviated by the overexpression of CsWAKL08, with OE3 exhibiting the greatest resistance, followed by OE1 and OE2. OE3 exhibited the smallest lesions, which were approximately 48% the size of those in the CK plants, with OE1 exhibiting a similar lesion size (49% of CK) and OE2 exhibiting slightly larger lesions (77% of CK) (Fig. 4g). The disease index, which was used to quantify disease severity, was decreased by 31% (OE2) to 58% (OE3) in the transgenic plants relative to the CK plants (Fig. 4h). The resistance of the transgenic plants was further evaluated by the infiltration method. At 10 dpi, canker symptoms were detected in the wild-type plants, whereas markedly reduced symptoms were observed in the overexpression plants (especially OE1) (Fig. 4i). These results therefore indicated that the overexpression of CsWAKL08 can strongly enhance resistance to Xcc in transgenic citrus.

### CsWAKL08 silencing confers CBC susceptibility

**Fig. 5:**
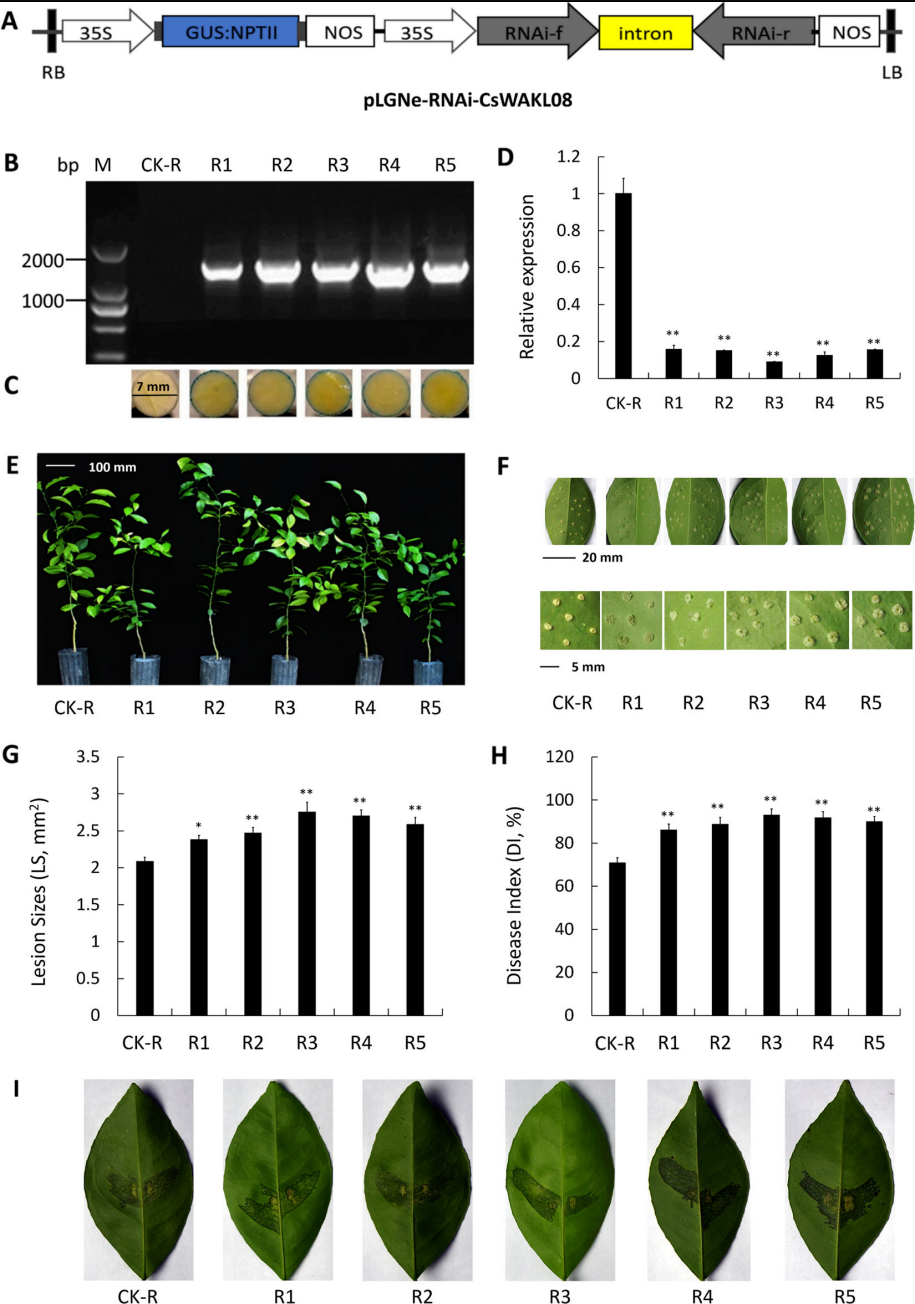
Assessment of Xcc responses in the CsWAKL08-silenced plants. **a** Plasmid used for RNAi assays; 35S, cauliflower mosaic virus 35S promoter; NOS, NOS terminator; NPTII, NptII gene; LB: left border; RB: right border. Transgenic plants were confirmed by PCR (**b**) and GUS staining (**c**). **d** CsWAKL08 expression as assessed via qRT-PCR with CsActin (GenBank: GU911361.1) as a normalization control. **e** Phenotypes of the RNAi transgenic plants. Scale bar = 100 mm. **f** Xcc-induced disease symptoms of the transgenic and CK plants at 10 dpi. The scale bar for the leaves was 20 mm, while for the lesions, it was 5 mm. For analysis of disease resistance, lesion sizes (LS) (**g**) and disease index (DI) (**h**) were analyzed. In (**d**), (**g**) and (**h**), *P < 0.05, significant difference; **P < 0.01, extremely significant difference; data are the mean ± SD. **i** CBC resistance assay of the RNAi plants using the infiltration method. Xcc-induced disease symptoms were observed at 10 dpi

To additionally assess the importance of CsWAKL08 in the Wanjincheng plants, we knocked down CsWAKL08 via RNA interference (RNAi). The RNAi sequence was digested and inserted into the pLGNe vector under the control of the CaMV 35S promoter (Fig. 5a). To verify the resultant transgenic plants, we performed PCR, and 5 RNAi plants (R1–R5) were obtained (fragment: 1456 bp) (Fig. 5b). These five plants were subjected to verification via GUS staining (Fig. 5c). The five plants exhibited relatively low CsWAKL08 expression relative to the CK plants (<20%), as validated by qRT-PCR (Fig. 5d). Compared to CK, R5 exhibited a relatively smaller size (Fig. 5e), which may be a result of grafting. The transgenic plants exhibited larger pustule eruptions relative to the CK plants (Fig. 5f). Therefore, we concluded that silencing CsWAKL08 significantly enhanced the susceptibility to CBC. Diseased lesions in the transgenic plants were significantly larger than those in the CK plants (114% (R1)—132% (R3)) (Fig. 5g). CBC severity analysis revealed that the mutant plants had significantly higher DI scores than the CK (Fig. 5h), with DI rising from 22% (R1) to 35% (R3). At 10 dpi of infiltration, the five RNAi plants showed more severe symptoms than the CK plants. Pustules were even observed at the infected sites (Fig. 5i). These data thus suggested that the silencing of CsWAKL08 in sweet orange increased the susceptibility to Xcc. As such, CsWAKL08 mutants exhibited increased CBC susceptibility, suggesting that CsWAKL08 is important for CBC resistance.

### CsWAKL08 engages the antioxidant system to reconstruct ROS homeostasis as a defense response to Xcc infection

**Fig. 6:**
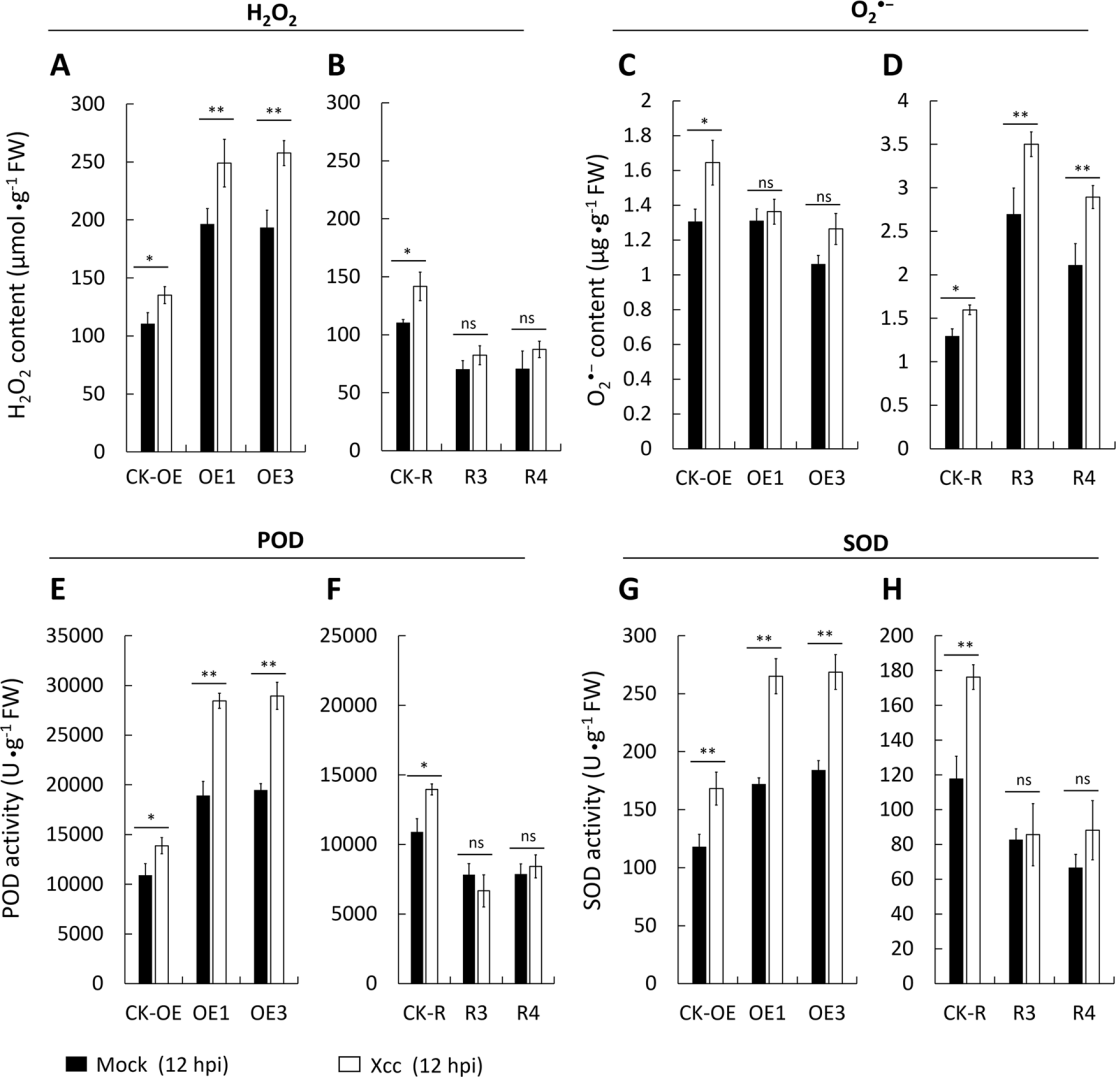
CsWAKL08 regulates ROS homeostasis in the transgenic plants. The levels of H_2_O_2_ (**a**, **b**), O_2_^•^− (**c**, **d**), POD (**e**, **f**) and SOD (**g**, **h**) in both the overexpression and RNAi plants were assessed at 12 hpi inoculated with mock (DDW) (filled bars) or Xcc (blank bars). *P < 0.05, significant difference; **P < 0.01, extremely significant difference; ns: no significance; Student’s *t*-test. Data are the mean ± SD

Plant antipathogen responses often depend on the production of excess ROS, which will induce oxidative damage to pathogens or plant apoptotic cell death^30–33^. To determine whether ROS homeostasis was involved in CsWAKL08-mediated resistance to Xcc, we analyzed the relative ROS levels in the CK and transgenic plants. The overexpression plants (OE1 and OE3) with higher CBC resistance and the RNAi plants (R3 and R4) with higher CBC susceptibility were chosen for this analysis. We found that the concentrations of H_2_O_2_ and O_2_^•^− were altered in these transgenic cells (Fig. 6a–d). Specifically, in the OE1 and OE3 plants exhibiting Xcc resistance, the H_2_O_2_ levels were higher than those in the CK plants (Fig. 6a), while O_2_^•^− was lower (Fig. 6c). The high H_2_O_2_ levels could cause hypersensitivity (HR) in the Xcc infection process. In R3 and R4 exhibiting Xcc susceptibility, the ROS concentrations were opposite to those of the overexpression plants (Fig. 6b, d). These results provide insight into a potential link between the ROS levels and Xcc resistance.

Plants have a well-developed system of antioxidant enzymes, including peroxidase (POD) and superoxide dismutase (SOD)^34,35^. As ROS are inducible and tightly regulated by CsWAKL08, we initially examined the expression of antioxidant enzymes to obtain additional evidence supporting this phenotype. Both the POD and SOD activities were upregulated by CsWAKL08 overexpression and inhibited by CsWAKL08 silencing (Fig. 6e–h). Compared with the H_2_O_2_ level, POD served as the H_2_O_2_ producer. The overexpression of CsWAKL08 enhanced the induction of antioxidant enzymes by Xcc infection, while CsWAKL08 silencing led to decreased induction (Fig. 6e–h). Thus, we were able to correlate the increased levels of resistance in the CsWAKL08-overexpressing cells with reconstructed ROS homeostasis, which was controlled by a more active enzyme system.

### CsWAKL08 positively regulates JA accumulation

**Fig. 7:**
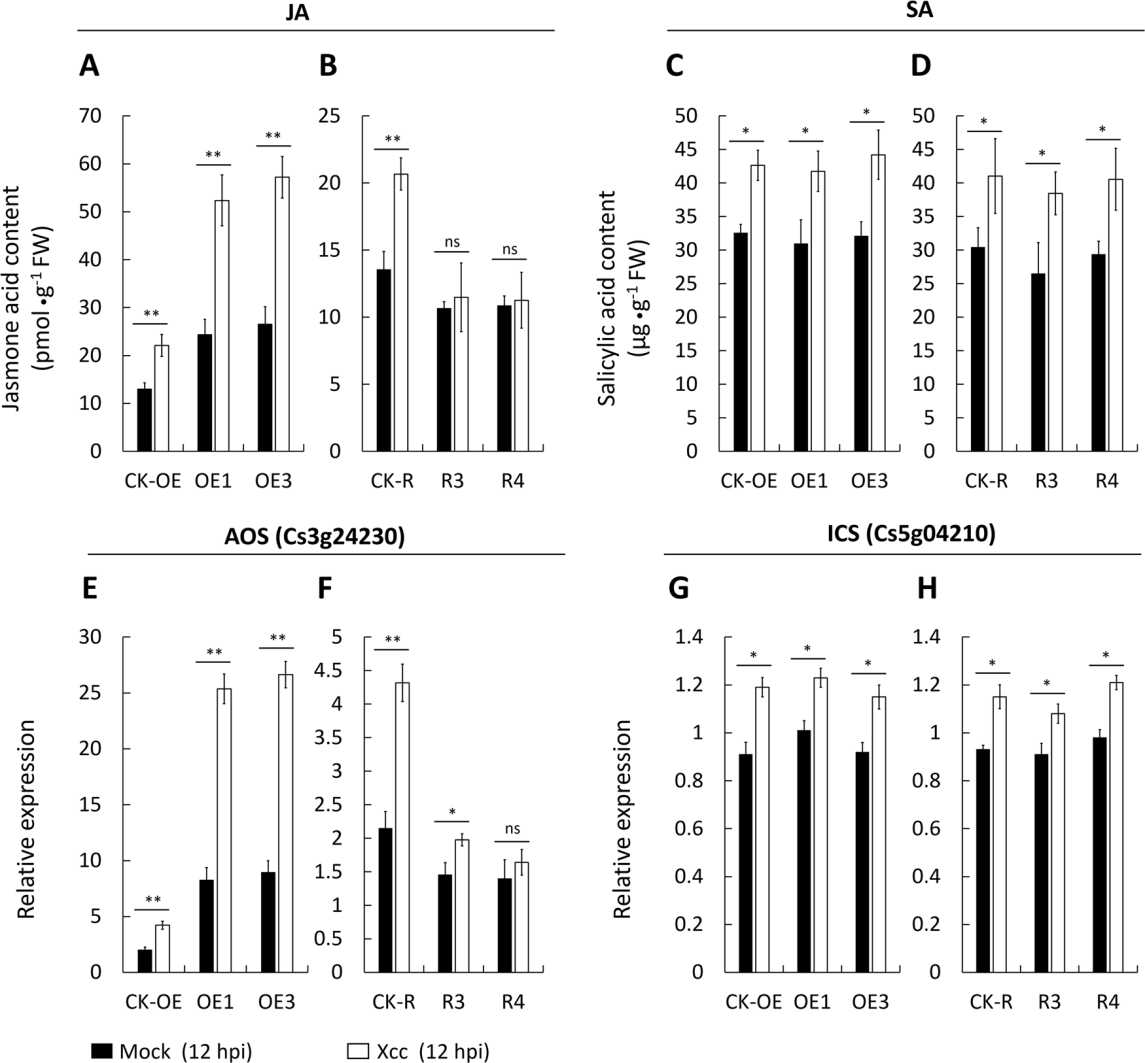
CsWAKL08 positively regulates JA biosynthesis in transgenic plants. The contents of JA (**a**, **b**) and SA (**c**, **d**) in both the overexpression and silencing plants were assessed at 12 hpi with mock (DDW) (filled bars) or Xcc (blank bars). The CsAOS (**e**, **f**) and CsICS (**g**, **h**) expression profiles in the overexpression and silencing plants assessed at 12 hpi with mock (DDW) (filled bars) or Xcc (blank bars) were detected by qRT-PCR with CsActin (GenBank: GU911361.1) as an internal control. *P < 0.05, significant difference; **P < 0.01, extremely significant difference; ns: no significance; Student’s *t*-test. Data are the mean ± SD

Taken together, our results suggest that CsWAKL08 may inhibit Xcc infection with innate immunity, thus reducing disease incidence. Phytohormones, including SA and JA, have been found to play pivotal roles in immune signaling networks^36–38^. In this study, we measured the SA and JA contents in our transgenic and CK plants. In addition, the transcript levels of the genes *CsAOS* (CAP: Cs3g24230) and *CsICS* (CAP: Cs5g04210) involved in JA and SA biosynthesis, respectively, were examined^39–43^. Compared to CK, OE1, and OE3 had significantly higher JA contents, which were upregulated sharply upon Xcc infection, whereas in R3 and R4, the JA contents were lower than those in CK and were insensitive to Xcc infection (Fig. 7a, b). In comparison to the JA contents, the SA contents were not significantly induced by either the overexpression or silencing of CsWAKL08 (Fig. 7c, d). Based on the hormone content results in the transgenic plants and CK upon Xcc infection, we concluded that CsWAKL08 positively regulates JA accumulation.

Consistent with these JA and SA measurements, the expression of CsAOS was sharply upregulated relative to that in CK in the CsWAKL08-overexpressing plants upon Xcc infection, whereas in the CsWAKL08-silenced plants, this expression was insensitive to Xcc induction (Fig. 7e, f). In contrast, the SA levels and the CsICS expression differed little between the CsWAKL08 transgenic and CK plants (Fig. 7g, h). These results indicate that CsWAKL08 may play a role in JA accumulation by regulating JA biosynthesis.

### CsWAKL08 regulates JA-dependent signaling in response to Xcc infection

**Fig. 8:**
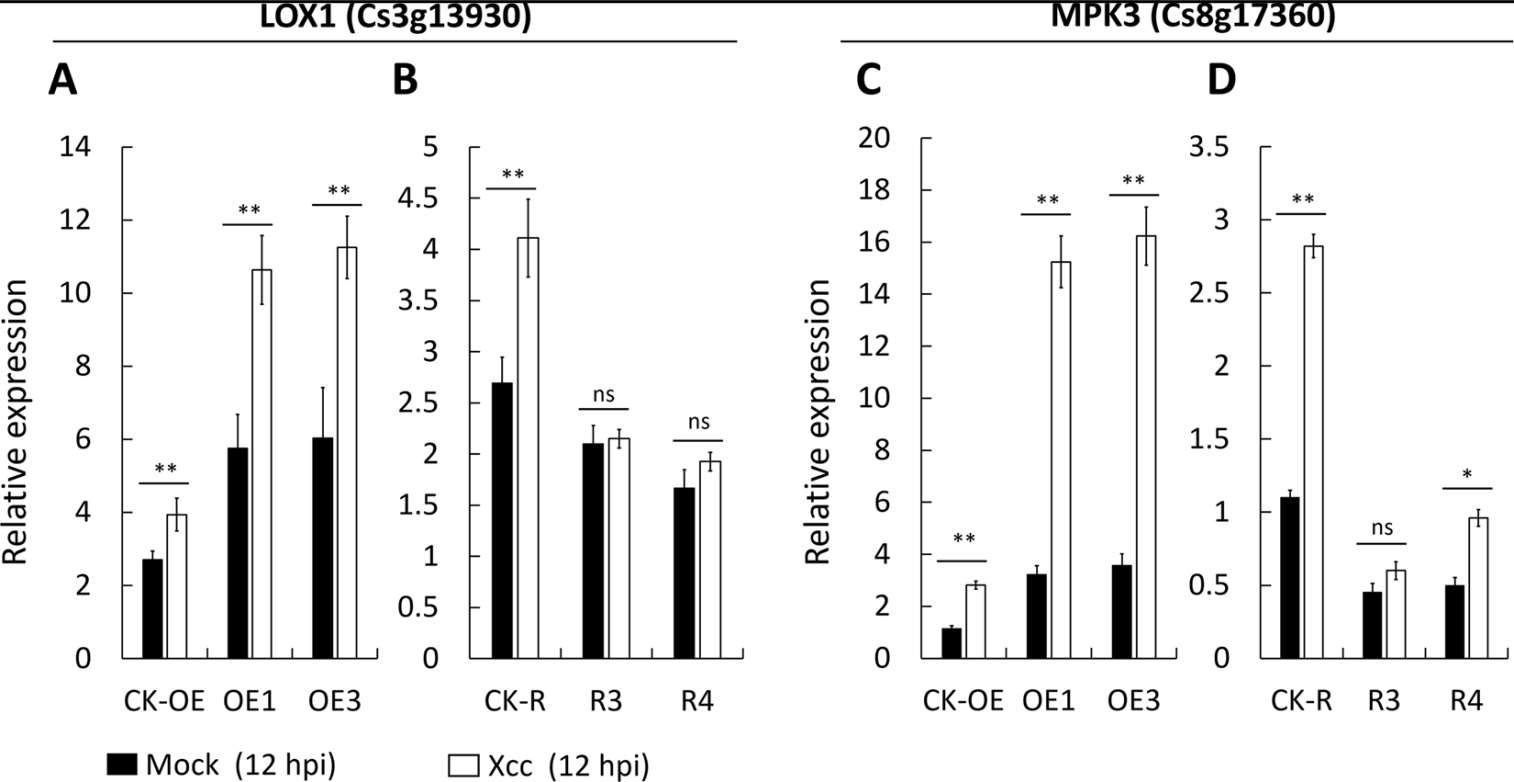
CsWAKL08 positively regulates the expression of the JA-responsive genes in the transgenic plants. CsLOX1 (**a**, **b**) and CsMPK3 (**c**, **d**) expression in the overexpression and silencing plants was assessed at 12 hpi with mock (DDW) (filled bars) or Xcc (blank bars) by qRT-PCR with CsActin (GenBank: GU911361.1) as an internal control. *P < 0.05, significant difference; **P < 0.01, extremely significant difference; ns: no significance; Student’s *t*-test. Data are the mean ± SD

Notably, JA was found to constitutively accumulate in the CsWAKL08-overexpressing plants, leading us to hypothesize that CsWAKL08 could regulate Xcc infection by regulating JA-dependent signaling. To validate this hypothesis, we evaluated the expression of certain JA-responsive genes in these transgenic plants. LOX1 (lipoxygenase 1) and MPK3 (mitogen-activated protein kinase 3) are PR proteins that have known roles in plant antipathogen defense responses and have been demonstrated to be involved in JA-dependent signaling^18,44^. As expected, CsLOX1 (CAP: Cs3g13930) was up- and downregulated in the CsWAKL08 overexpression and silencing plants, respectively (Fig. 8a, b). With respect to Xcc induction, *CsLOX1* expression was sharply upregulated in these plants relative to the CK plants (Fig. 8a), whereas in the CsWAKL08-silenced plants, this Xcc-mediated induction was reduced (Fig. 8b). With respect to CsMPK3 (CAP: Cs8g17360), similar to CsLOX1, its expression was also significantly upregulated by CsWAKL08 overexpression and Xcc-inducible expression (Fig. 8c), whereas CsMPK3 expression in the CsWAKL08-silenced plants was less significantly increased upon Xcc infection (Fig. 8d). This result suggests that CsWAKL08 plays roles in basal disease resistance. Based on our analysis of the JA-responsive genes in the transgenic plants after Xcc inoculation, we concluded that CsWAKL08 can regulate Xcc infection by regulating JA-dependent signaling to activate JA responses, thereby conferring CBC resistance and tolerance.

## Materials and methods

### Comprehensive annotation and bioinformatics analysis of the *CsWAKL* genes

To exhaustively identify sweet orange *CsWAKLs*, we downloaded the proteome and genome of *C. sinensis* from the CAP^26,45^ as a data source. *CsWAKL* searches were performed using all 27 *WAK* and *WAKL* genes previously reported in *A. thaliana* as the BLAST query^15^. Exhaustive data mining and annotation were executed via a semiautomated three-step expert process that should avoid the errors inherent in fully automated predictive methods^32,46^. During the annotation process, the gene reannotation tool Fgenesh++^47^, Citrus genomic variation database (CitGVD)^48^, as well as the protein functional domain analysis tools Pfam V32^49^ and SMART^50^, were used for the functional reannotation, while Scipio^51^ was used to retrieve *CsWAKLs* misannotated by the automated processes. Sequences that had both kinase- and extracellular-related domains in the predicted protein were selected as the putative *CsWAKL* genes^25^. Putative sweet orange WAKLs were designated as ‘CsWAKL’, followed by a number indicating their chromosomal order. The neighbor-joining phylogenetic tree was constructed with MEGA V7.2^28^ with the full-length protein sequences aligned by Clustal X^52^ and BioEdit V2.0^53^. The gene structures of *CsWAKLs* were visualized with GSDS 2.0^54^, while conserved motifs were detected with MEME V5.1^29^. SignalP 4.0^55^ and CELLO^56^ were used for the prediction of SPs and subcellular localization.

### Plant, bacterial materials, and treatments

Plant materials in this study were sampled from the National Citrus Germplasm Repository, Chongqing, China. Wanjincheng (*C. sinensis*) and Calamondin (*C. madurensis*) were sampled for the CBC assay, hormone treatment, and other experiments, while genetic manipulation of the Wanjincheng plants was additionally conducted. The plants were grown in a 28 °C greenhouse. The XccYN1 Xcc variants were collected from naturally infected sweet orange leaves from Yunnan Province in China. The Xcc bacteria were cultured at 28 °C in peptone-yeast extract-malt extract (PYM) containing D-glucose: 1.5% (w/v) and appropriate antibiotics^23^. For analysis of CsWAKL08 expression dynamics, healthy leaves were freshly collected and then treated with a 1000-fold dilution of Xcc (OD600 = 0.5) and incubated under the same conditions as above. At 0, 6, 12, 24, 36 and 48 hpi, samples were then collected for the Xcc assays. Exogenous hormone assays were conducted using qRT-PCR with leaf samples that were treated using 10 μmol/L SA or 100 μmol/L MeJA and ABA.

### Assessment of CsWAKL08 subcellular localization

The CDS of CsWAKL08 (without a stop codon) was amplified with primers F_SC_ (CGGGGTACCATGGCTGTTCATCAACATTATCTGG) and R_SC_ (TCCCCCGGGTCACTGGTTTGAAATTAAAGGATCT). This sequence was then cloned into the pLGNe-GFP vector, yielding the pLGNe-CsWAKL08-GFP vector. Next, *Agrobacterium tumefaciens* EHA105 containing the pLGNe-CsWAKL08-GFP vector was used to infect onion epidermal cells for 48 h, after which GFP fluorescence was observed by microscopy (Olympus, Japan).

### Construction of the overexpression and RNAi plasmids

For generation of the overexpression plasmids, the full-length CsWAKL08 CDS was amplified using primers F_OEC_ (CGGGGTACCATGGCTGTTCATCAACATTATCTGG) and R_OEC_ (CGGAATTCTCACTGGTTTGAAATTAAAGGATCT) and inserted into the vector pGLNe with the CaMV 35S promoter. For generation of the RNAi vectors, primers F_RIC_ (GCTCTAGAGGCGCGCCCCTACTCAGAAATCCGGTTGC) and R_RIC_ (CGGGATCCATTTAAATCTGAACTTAGTTGCATTAA) were used to amplify a 304 bp fragment, which was integrated into the vector pUC-RANi. Then, the RNAi sequence was inserted into pLGNe to generate the final vector.

### Production and characterization of the transgenic plants

Heat shock was used to introduce these overexpression and RNAi plasmids into *A. tumefaciens* EHA105. Wanjincheng shoot segment transformation was conducted with *A. tumefaciens* via the method described by Peng^24^. PCR and GUS assays were used to confirm the transgenic plants. Primers F_OED_ (CGACACGCTTGTCTACTCCA) and R_OED_ (TCACTGGTTTGAAATTAAAGGATCT) were used for the overexpression plants, while primers F_RID_ (TGCAACTAAGTTCAGATTTAAATGTGTAA) and R_RID_ (ATTCAAGTCGGATCCAAATACCTGCAAA) were used for the silencing plants. The GUS activity in the transgenic plants was assessed via a histochemical procedure^57^. qRT-PCR was performed to analyze the expression of CsWAKL08 in the transgenic plants. Plants with empty vectors served as controls in the PCR, GUS, and qRT-PCR assays.

### Analysis of CBC resistance in the transgenic plants

The Xcc resistance of the transgenic plants was assessed in vitro as described previously^24^. Briefly, six punctures were made in each of six mature healthy leaves per transgenic plant with a 0.5-mm-diameter pin, and then, 1 µL of XccYN1 bacterial suspension (1 × 10^8^ CFU mL^−1^) was inoculated in these sites. The development of CBC disease was studied at 10 dpi. Disease index (DI) values and disease lesion area values were used to assess resistance to Xcc. DI was calculated with the formula previously reported by Peng^24^. The resistance of the transgenic plants was further evaluated by infiltration of XccYN1 bacterial suspension (1 × 10^8^ CFU mL^−1^). The canker symptoms were photographed at 10 dpi.

### Measurements of ROS contents in the transgenic plants

To confirm the physiological effect of CsWAKL08 in the transgenic plants compared with the CK plants after mock or Xcc inoculation at 12 hpi, we analyzed the concentrations of H_2_O_2_ and O_2_^•^− using commercial kits. All leaf samples were frozen using liquid nitrogen and ground into a fine powder for measurement. The concentrations of H_2_O_2_ and O_2_^•^− and the activities of POD and SOD were measured and defined using a corresponding commercial kit (SinoBestBio, China) based on the provided directions. The tests were repeated three times.

### Evaluation of SA and JA contents in the transgenic plants

For confirmation of CsWAKL08-associated hormone induction, SA and JA were extracted from the leaves of the transgenic plants and the CK plants to measure the SA and JA contents. Leaf samples (1 g fresh weight) were collected and frozen in liquid nitrogen, ground into fine powder, and sequentially extracted with 80% methanol overnight followed by centrifugation at 13,000 r min^−1^ for 10 min. The supernatants were then evaporated and resuspended using 1% acetic acid. Oasis cartridges (Waters, USA) were used to purify the hormones based on the provided directions, with 10% methanol then used to dissolve these hormones before HPLC analysis. The tests were repeated three times, and the standard error was calculated.

### RNA isolation, cDNA synthesis, and qRT-PCR

Total RNA was isolated from ground tissue samples with a kit (AidLab, China) based on the provided directions. Reverse transcription was then performed with a kit (TaKaRa, China), and qRT-PCR was conducted with a kit (Bio-Rad, USA) and QuantStudio 7 (Applied Biosystems, USA), with citrus actin serving as an internal control (GenBank: GU911361.1) using the primers F_Actin_ (CATCCCTCAGCACCTTCC) and R_Actin_ (CCAACCTTAGCACTTCTCC). The qRT-PCR program was as follows: 95 °C predenaturation for 5 min, followed by 40 cycles at 95 °C for 10 s and 56 °C for 30 s. A 20 μL reaction mixture contained cDNA (100 ng), primers (0.5 μM) and PCR mix (10 μL). The 2^−∆∆CT^ method was used to assess relative gene expression^58^. Primers used for qRT-PCR (Table S3) were designed with the NCBI Primer BLAST program. For each gene and each sample, triplicate biological and triplicate technical replicates were analyzed.

### Statistical analysis

All statistical testing was conducted using SPSS V22 (IBM, USA). Data differences were compared via analysis of variance (ANOVA) with Fisher’s LSD test, with *P < 0.05 as the threshold of significance and **P < 0.01 as the threshold of extreme significance. Data are the mean ± SD.

## Discussion

### The expression of CsWAKLs can be induced by bacterial pathogens

WAKLs have been shown to be important in disease resistance^2,3,18,59^, phosphorus starvation tolerance, and developmental processes such as root growth^60^. Multiple previous studies have shown that WAKLs are transcriptionally regulated during bacterial infections^19,61,62^. In our analyses of Xcc-infected species, WAKLs were consistently overrepresented. In this study, we identified the WAKL family via systemic annotation (Fig. 1a) and then investigated the regulation of CsWAKLs early in the Xcc infection process, confirming that three *CsWAKL* genes (*CsWAKL01*, *CsWAKL08*, and *CsWAKL20*) are differentially expressed upon Xcc infection in CBC-resistant and CBC-sensitive varieties (Fig. 1d, e).

### CsWAKLs are essential for CBC resistance

The importance of WAKs for plant disease resistance was initially recognized indirectly from studies using WAK mutants that demonstrated altered triggering of defense-related responses^63^. Since these initial findings, many studies have highlighted the role of the WAK genes in pathogen resistance. In dicots, such WAKLs include RFO1/WAKL22^64,65^, AtWAK1^66^ and SlWAK1^62^. In rice, OsWAK1 has been shown to improve *M. oryzae* resistance^59^. Recent studies have also identified specific QTLs encoding WAK genes that are linked to maize fungal resistance^3,18^. WAKs are plasma membrane receptors with N-terminal cysteine-rich galacturonan-binding regions and C-terminal kinase domains^16,67^. In the context of pathogen invasion, fragmented pectins generated by pathogen activity or wounding are recognized by these WAKs as ligands^19^. WAKs then transduce this signal into the cell and activate downstream genes, leading to the induction of stress responses^67^. Our work highlighted three CsWAKLs involved in Xcc infection and further extended the list of known WAKLs, confirming a role for WAKLs in pathogen immunity in sweet orange. CsWAKL08 was demonstrated in this study to have a classic 3-domain structure (Fig. 2c) and plasma membrane localization (Fig. 3), and we explored its functional role in depth using overexpression and RNAi silencing strategies. We found that CsWAKL08 overexpression conferred CBC resistance, whereas CsWAKL08 silencing conferred CBC susceptibility (Figs. 4, 5).

### CsWAKL08 participates in plant defense responses via controlling JA signaling and ROS homeostasis

Phytohormones, including SA and JA, have been shown to play pivotal roles in innate immune signaling^36–38^. Indeed, Zuo et al.^18^ provided evidence that AOS is slightly upregulated by ZmWAK in response to pathogen infection. Consistent with this result, we observed a link between CsWAKL08 transformation and JA signaling, similar to these previous findings in maize. In contrast to ZmWAK, we did not detect SA elevation in the cells overexpressing CWAKL08 (Fig. 7), suggesting that these WAKLs have related but distinct defense mechanisms.

The oxidative burst, particularly the production of H_2_O_2_ and O_2_^•^−, is a common innate response in plant cells upon pathogen detection^34^. H_2_O_2_ can drive JA biosynthesis via the octadecanoid pathway, leading to the generation of a wide array of JA-associated genes and products that regulate secondary metabolite production. Measurement of ROS production and defense gene expression in the CsWAKL08-overexpressing plants confirmed a role for this gene in the ROS-associated pathogen responses, consistent with the loss of resistance observed in the RNAi plants (Fig. 6). There is relatively little evidence to suggest that WAKLs can directly or indirectly modulate PR gene expression^34^. Plants overexpressing CsWAKL08 displayed enhanced expression of JA-responsive PRs, which may be due to the regulation of the JA-dependent signaling pathway.

Based on our findings, we speculate that CsWAKL08 plays a crucial role in defending against CBC primarily via regulation of the ROS levels and through the activation of PRs via JA-dependent signaling. These findings suggest links among WAKL-mediated quantitative disease resistance, the JA signaling pathway, and ROS homeostasis.

## Supplementary information

Supplementary Figures

Supplementary Tables
