# Supplementary material for: CsWAKL08, a pathogen-induced wall-associated receptor-like kinase in sweet orange, confers resistance to citrus bacterial canker via ROS control and JA signaling
Source: Hortic Res. 2020 Apr 1;7:42. doi: 10.1038/s41438-020-0263-y (PMC7109087; doi:10.1038/s41438-020-0263-y)

**Fig. S1 Different expression profiles of 21 CsWAKLs induced by Xcc.**

Samples collected from Wanjincheng (blue bars) and Calamondin (orange bars) at 0, 6, 12, 18, 24 36 and 48 hpi of Xcc infection. Data are means ± SD from three biological replications.


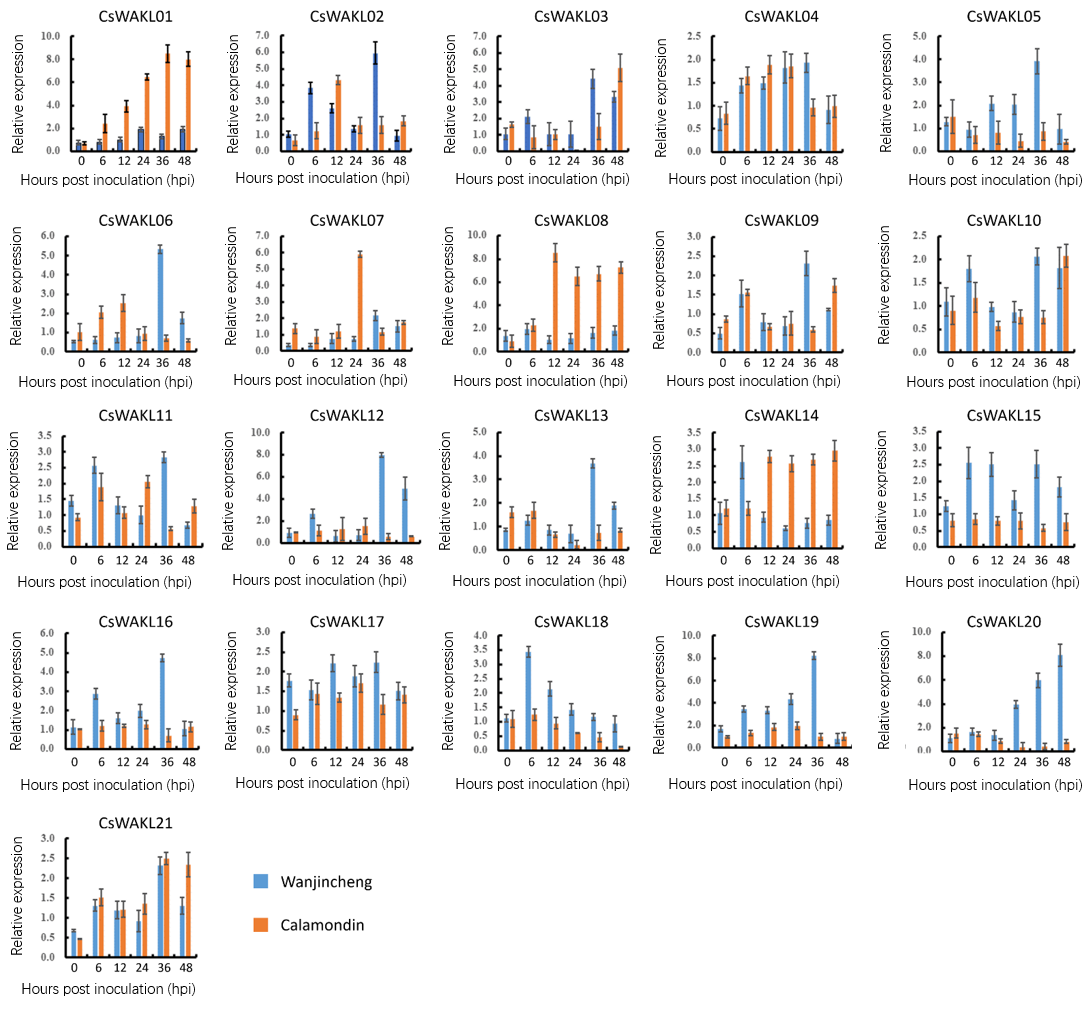

Supplement: Supplementary file 1 — Supplementary Figures [file 41438_2020_263_MOESM1_ESM.docx]
